# Understanding community and health system acceptability, readiness and perspectives on the introduction of new vector control approaches for malaria control in Papua New Guinea

**DOI:** 10.1186/s12936-026-05990-1

**Published:** 2026-06-16

**Authors:** Zebedee Kerry, Rachael Farquhar, Kilon Manuv, Alma Auwun, Petrina Johnson, Paul Daly, Michael Macdonald, Stephen Bell, Maria Ome-Kaius, Stephan Karl, Moses Laman, Leanne J. Robinson

**Affiliations:** 1 Burnet Institute, Melbourne, Australia; 2 Papua New Guinea Institute of Medical Research, Goroka, Papua New Guinea; 3 James Cook University, Townsville, Australia; 4 Public Health and Preventive Medicine, Monash University, Melbourne, Australia; 5 Innovative Vector Control Consortium, Liverpool, England

**Keywords:** Malaria, Vector borne diseases, Implementation research, Vector control tools, Residual spraying, Community acceptability, Socio-cultural influences

## Abstract

**Background:**

Malaria remains a major public health challenge in Papua New Guinea (PNG) and accounted for most malaria cases and deaths in the Western Pacific Region in 2023. Despite current national efforts centred on insecticide treated nets (ITNs), there is a pressing need to explore supplementary vector control tools, given the resurgence of malaria in PNG since 2015, partly driven by declining insecticidal efficacy of ITNs. The Newly Adapted Tools and Network Against Mosquito Borne Disease Transmission (NATNAT) project is assessing the feasibility and acceptability of complementary vector control approaches in PNG communities and the health system. These tools include residual spraying, spatial emanators, and larval source management.

**Methods:**

We conducted an exploratory qualitative study to examine community acceptability, readiness and perceptions of current vector control tools such as ITNs and the potential reintroduction of supplementary vector control approaches, like residual spraying. Using in-depth interviews and focus group discussions, data were collected from four villages along the north coast of Madang Province: Megiar, Bulal, Mirap, and Wasab. Transcripts were analyzed thematically using inductive techniques, with collaborative review to refine key themes by study team.

**Results:**

The current malaria control practices and contextual factors shaped community perceptions and the prospective acceptability of residual spraying. Community members demonstrated a strong understanding of malaria risk, including seasonal transmission patterns and breeding sites, and reported both traditional and program-implemented vector control approaches used in their village. Engagement for vector control activities was motivated by strong leadership, personal values, and a comprehensive understanding of malaria risk factors. Community support for supplementary vector control measures, alongside a strong desire to address existing gaps in mosquito protection, were key drivers of acceptability. Addressing insecticide safety concerns informed in part by historical DDT spraying campaigns experiences through tailored, context-specific communication strategies will be critical for the successful adoption of residual spraying. Affordability and uptake of new vector control tools into existing programs are essential for sustainable uptake into policy and practice.

**Conclusions:**

Findings demonstrate the prospective acceptability of new vector control tools in PNG and underscores the importance of sustained, in-depth community engagement. To ensure effective implementation, policy development must consider community norms and practices, societal attitudes, and resource availability.

**Supplementary Information:**

The online version contains supplementary material available at 10.1186/s12936-026-05990-1.

## Background

Malaria remains a significant public health challenge in Papua New Guinea, which accounted for 65.6% of total malaria cases and over 78.2% of malaria-related deaths in the Western Pacific Region in 2024 [1]. The country has an established National Malaria Vector Borne Disease Program (NMVBDP) which works closely in partnership with funders, implementers, research organisations and sub-national health teams [2]. Currently, insecticide treated nets (ITNs) are the only programmatically implemented vector control tool distributed nationally through the NMVBDP, with 1.3 million ITNs provided free of charge in 2023 [3].

Since 2004, with the financial support from the Global Fund to Fight AIDS, Tuberculosis and Malaria, PNG scaled up its malaria control program with 3-yearly nationwide distribution of ITNs that enabled ITNs to be widely distributed to households [4]. While this initial rollout of ITNs coincided with a decline in the malaria burden, a resurgence of malaria has been observed since 2015 [3].Key contributing factors to this resurgence were the reported reduction in the insecticidal efficacy of ITNs distributed in 2013 and 2019 [3], low usage rates for ITNs despite increased coverage [5–7] and early and outdoor biting behaviours of vectors resulting in a decreased protective efficacy of ITNs as more exposure to mosquitoes occurred before individuals were protected under the net [8].

The acceptability of program-implemented vector control tools such as ITNs and novel vector control approaches such as insecticide treated plastic sheeting have previously been explored in PNG [9, 10]. Factors shaping ITNs use include household sleeping arrangements and adequate ITN coverage, ITN maintenance and household knowledge on ITN usage to prevent malaria infection [11]. For novel approaches, such as insecticide-treated plastic sheeting, variation in housing structures often complicated the installation process alongside, whilst concerns about possible side effects of insecticide treated plastic sheeting and apprehension about not being present during installation further reduced acceptability [9]. Despite these barriers, perceived effectiveness of the insecticidal properties and protection against malaria supported high acceptance amongst community members [9]. Recognizing the limitations of ITNs as a standalone intervention and complex, context-specific factors influencing the community uptake of new vector control tools, the Newly Adapted Tools and Network Against Mosquito Borne Disease Transmission—NATNAT partnership is investigating the feasibility and acceptability of supplementary vector control tools such as residual spraying, spatial emanators and larval source management within communities and the PNG health system.

Residual spraying involves the application of residual insecticides to indoor walls, ceilings and sheltered outdoor structures where malaria vectors may come into contact with the insecticide while seeking hosts [12, 13]. The acceptability of residual spraying varies significantly across different settings, with household acceptance rates ranging from 97.6% in Southern Mexico to as low as 27.8% in eastern Ethiopia [14, 15]. Residual spraying has a historical precedent in PNG, having been introduced in the 1930s with its use intensifying throughout the late 1950s to 1970s as a malaria control strategy [16, 17]. Despite its demonstrated effectiveness in reducing malaria prevalence, residual spraying was eventually discontinued in the 1980s. The use of dichlorodiphenyltrichloroethane (DDT) in residual spraying operations raised concerns about environmental and domestic animal health, an increase in rat populations, and a rise in bedbug infestations [18]. These issues contributed to widespread community resistance and rejection of residual spraying as a viable malaria control strategy [18]. In addition, challenges with, workforce capacity for training, transport, operational limitations, limited supervision and assistance for implementation and funding led to the malaria program stopping IRS operations in 1983 [19–21].

Alternative vector control tools such as spatial emanators and larval source management are also promising options for integration into the PNG’s vector control strategy. Spatial emanators have evolved over time, from formats that require frequent replacement and external energy sources for active emanation, to formats that passively dispense the active ingredient for months [22]. Potentially filling a current gap in protection, the programmatic use of spatial emanators has been explored across many countries such as Cambodia, Tanzania and Haiti [23, 24]. Larval source management has been utilised as an effective intervention to supress mosquito populations in urban development areas [25, 26] and within rural communities [26, 27]. A recent narrative review of countries that previously eliminated malaria, noted 73 of the 85 countries reporting vector control efforts used some form of LSM [26]. In PNG, larval source management is currently being used in the mine impact zone on Lihir Island as part of the services provided by Newcrest Mining Ltd [28] and other private sector workforce protection programs [29]. To support broader implementation across provinces, it will be important to explore opportunities for community-led expansion of larval source management.

Supplementary vector control approaches are often labour-intensive, behaviour dependent and require ongoing support—meaning the long-term success hinges on strong community engagement and ownership [30]. Across the Pacific, locally driven models have demonstrated how empowering communities to co-design vector control can enhance sustainability. In Vanuatu, leveraging existing social networks and integrating malaria activities within broader health and disease priorities through locally appropriate multi-level media campaigns improved community participation and collective responsibility [31]. In PNG, initiatives such as the Batri Net program, have shown that when community leaders drive decision-making and engagement strategies align with local values and communication norms, uptake increases [32]. A systematic review of community participation for malaria elimination further reinforces that while top-down coordination is necessary for scale, long term success requires bottom-up leadership. Blended models that pair programmatic efficiencies with pathways for community governance offer a more realistic route to sustained implementation and ownerships [33].

To date, limited research has been conducted on the acceptability, readiness and uptake of supplementary vector control tools, including residual spraying, larval source management and spatial emanators within the PNG context [34]. This study investigates community perceptions and contextual factors that may influence the acceptability and uptake of new vector control tools, particularly residual spraying across four communities along the North Coast of Madang, PNG, where a residual spraying implementation research study was being planned. It also explores how broader social and health systems factors shape community perceptions and their readiness to adopt these novel vector control approaches [35].

## Methods

### Author positionality

Data collection was undertaken by 4 researchers (ZK, RF, KM, AA). ZK, KM, AA are Papua New Guinean researchers from the PNG Institute of Medical Research, based in Madang and spent ample time in the communities during data collection. ZK and KM are male researchers, while AA and RF are female researchers. ZK, KM, and AA have backgrounds in Social Science research and Health Management. RF is Australian with a background in Social Science and Health Systems Research and spent time in Madang throughout the data collection period. All four researchers hold relevant degrees and have experience in social science, health management, qualitative research, and public health. At the time of data collection, all 4 researchers had been working together for ~ 3 years so collaborative reflexivity [36] was regular given ongoing, trusted relationships were established. This meant ZK, KM, AA, and RF often engaged in reflexivity together rather than alone, having a team discussion on data collection, interpretation, and analysis, asking each other questions about assumptions and decisions made throughout different processes.

### Study design

This was an exploratory qualitative study designed to explore community acceptability and perceptions of current vector control tools such as ITNs and the community and health systems readiness for reintroduction of alternative vector control approaches such as residual spraying. It was conducted between 27th August 2021–14th October 2021. The study was guided by an interpretive approach that emphasised the need to access locally situated, subjective understandings of social life and social phenomena [37]. Assessing local understandings of vector control tools and residual spraying contributes to the study aims by identifying the factors of acceptability and health systems readiness that improve local uptake of new vector control tools and embeds sustainable implementation efforts into vector control programs. The description of this methods section was guided by the COREQ checklist (see supplementary information file 1).

### Study setting

Community level data collection was conducted in four villages; Megiar, Bulal, Mirap and Wasab, situated along the north coast of Madang province where field studies of residual spraying were planned. These four villages had long-standing relationships with the research team and over 10 years of epidemiological and transmission data to inform the implementation of complementary vector control tools. Two of the villages are inland (Wasab and Bulal), with 70 and 103 households respectively; two are coastal villages (Megiar and Mirap), with 180 and 208 households respectively. The four villages experience heterogeneity in vector composition, abundance and distribution resulting in malaria transmission variability and challenging malaria control program efforts towards elimination [8]. These coastal villages depend on fishing for both subsistence and income. The inland villages depend on subsistence farming including taro, cassava, banana, and yams for their livelihoods. The primary source of income includes cocoa and coconut (copra) production. Furthermore, many households rely heavily on the trade with betelnut (Areca catechu) and in some cases, it is the only cash crop grown, and produces a crucial source of income for many households.

Sub-national data collection was conducted across 6 provinces with established PNGIMR-Provincial Heath Authority partnerships in Vector Borne Disease Research: Madang, Western, New Ireland, West Sepik, NCD and Morobe provinces [38, 39]. These 6 provinces were selected to capture a range of settings with differing vector control capacity, operational experience and intervention coverage.

### Population, sampling and recruitment

Table 1 summarizes the number of interviews and focus group discussions conducted across the four villages and at the subnational level. A total of 24 in-depth interviews and 8 focus group discussions involving 53 participants.

**Table 1:** Summary of community and sub-national level interviews (SSIs) and focus group discussions (FGD)

Data collection methodology	Number of interviews per village—community level	Number of interviews per province—sub-national	Total no. of interviews
In-depth interviews	Megiar: 2 (2 Male) Wasab: 3 (2 Male, 1 Female) Mirap: 3 (2 male, 1 Female) Bulal: 3 (2 Male, 1 Female)	Madang Province: 2 Western Province: 1 NCD: 2 New Ireland Province: 3 West Sepik Province: 2 Morobe Province: 2	24
Focus group discussions	Megiar: 2 (7 Female, 7 Male) Wasab: 2 (6 Female, 6 Male) Mirap: 2 (6 Female, 7 Male) Bulal: 2 (7 Female, 7 Male)		8 (53 participants)

At the community level, participants were recruited to participate in in-depth interviews and focus group discussions. Inclusion criteria for participation were: 18 years of age or older; residence in one of the four communities; willing and able to provide written consent to participate. During consent procedures, the study’s objectives were discussed with the participant. Sampling and recruitment strategies differed between in-depth interviews and focus group discussion. Purposive sampling [40]—defined as the involvement of deliberate participants who possess specific characteristics relevant to the studies objectives—was used to recruit participants from all four villages into in-depth interviews. This ensured breadth of socio-demographic characteristics (i.e. by gender, profession) and inclusion of peoples typically excluded from research due to, for example, education, literacy or disability. The four communities are characterised by varying literacy levels and participants were selected to reflect this diversity. Qualitative researchers (ZK, KM, AA, RF) worked with community leaders and community guides to identify community members that met the inclusion criteria. Interview participants included community leaders, heads of households, influential community members, mothers and fathers and youth. All invited participants agreed to take part and remained engaged throughout the study with no refusal to participate.

Participants were recruited using convenience sampling [40], defined as the selection of target population that meet certain practical criteria, such as easy accessibility, geographical proximity, availability at a given time, or the willingness to participate are included for the purpose of the study. This method was used to recruit available community members into gender-based focus group discussions. With the support of well-respected community guides from each village, participants were identified for recruitment. Recruitment took place in the community the day prior to data collection. The disadvantages of using guides meant at times, they recommended individuals they believed best met the ‘criteria’ for participation. This often resulted in the exclusion of people with lower levels of education or literacy, as well as those living with disabilities. To address this, the research team then actively sought to ensure that these individuals were not overlooked and had equal opportunity to participate in focus group discussions, by recruiting participants independently of the community guides. This strategy was effective in reaching individuals who may not otherwise have been identified and contributed to a broader range of perspectives in the discussions.

At the sub-national level, purposive sampling was used to identify and recruit key informants with research, government, implementation and private sector expertise related to vector control in PNG. These individuals were identified from investigators professional networks and invited to participate by email with a follow up interview arranged via zoom or in-person.

### Data collection

Qualitative data collection occurred prior to commencing residual spraying in the two intervention villages. Two main data collection methods were used: in-depth interviews and focus group discussions. Interviews used a semi-structured discussion guide to explore themes including strengths and weaknesses of current vector control tools, community views on vector control practises and impact and community views on the introduction of new vector control tools. Focus groups used a semi-structured discussion guide to explore collective views and meanings that lie behind the acceptability and introduction of new vector control tools, cultural beliefs that influence human behaviour towards vector control and barriers and enablers to specific uptake and use of vector control methods within a community.

Following each day of data collection, all researchers wrote individual memos in a shared document and participated in group debriefs. These sessions were used to reflect on assumptions, potential biases and decisions made during the research process. They were also used to discuss data saturation by reviewing emerging theories, assessing repetition across interviews and deciding whether further data collection was needed to ensure depth and variations in perspectives. The qualitative research team also regularly examined the power dynamics between themselves and community members.

Interviews and focus groups were conducted primarily in Tok Pisin, by a member of the qualitative research team in a communal area in the community that was deemed as safe for participants, offering audio privacy, and at a time convenient to participants. Cultural norms in the study communities often constrained women from speaking openly and freely in the presence of men, including the male researchers (ZK, KM). To address this, female researcher AA facilitated the female focus group discussions, fostering a culturally acceptable environment that encouraged more transparent and meaningful participation. Interviews lasted between 58 and 93 min and focus group discussions lasted for 58–94 min and were conducted in Tok Pisin language. Recordings were audio-recorded, transcribed, translated and de-identified ready for analysis.

### Analysis

Transcripts were uploaded into NVivo 12 in preparation for inductive analysis, and were coded thematically following Braun and Clarkes system for thematic analysis [41] following the 6 stage process, comprising: (1) familiarisation with the data, (2) generation of initial codes, (3) searching for themes, (4) reviewing themes, (5) defining and naming themes, and (6) producing and writing up the report. Three qualitative researchers (KM, ZK & RF) familiarised themselves with the data, and KM generated initial codes. Themes within the codes were then collated by the three researchers KM, ZK & RF. These themes were reviewed and refined through a collaborative process with the study team [41]. The preliminary findings of this study were shared with community members and stakeholders, including research team members and malaria program managers, through community awareness and engagement sessions. These findings informed prospective IRS activities across the 4 villages in Madang Province, as well as an IRS pilot in New Ireland Province, bringing insights from the community into planning discussions for community engagement strategies, communication messaging on IRS safety. Based on the findings, community awareness and engagement activities were subsequently conducted in the evening and at night rather than during the day. Information, Education and Communication (IEC) and Behaviour Change Communication (BCC) materials were also developed to support effective community engagement, address safety concerns, and promote informed acceptance of IRS activities.

### Ethical considerations

This study was approved by the PNG IMR Institutional Review Board (#2005), the Medical Research Advisory Committee in PNG (#20.19) and Alfred Hospital Ethics Committee (#67898) in Melbourne, Australia. Key ethical standards adhered to during the study included: obtaining written informed consent from all participants. Participants with limited literacy, who were unable to provide a written signature, indicated their consent by making an 'X' mark on the consent form; an independent literate witness from the community, not affiliated with the research team, co-signed the form to confirm that the participant had been fully informed and that their consent was voluntarily given. Participant anonymity and confidentiality were adhered to by de-identifying all interview and focus group discussion transcripts; and secure storage of research data on a password-protected computer.

## Results

### Community perspectives and drivers of vector control

#### Experiences of living in a high burden malaria context

Across the four villages, community members shared their common experiences with malaria. The impact of malaria was evident in its far-reaching effects on health, wellness, and productivity within their families and the community.

Many times, we can’t do work when our blood is weak because when malaria enters the body it weakens and disturbs the body so we can’t do work. That’s how we see and understand and mostly we have realized that malaria is like a poison that also kills people. If we did not seek treatment quickly at the health facility, we realize that people will die.—Community Member, Female

Participants highlighted the vulnerability of children and elders to malaria. Risk for malaria transmission varied during particular seasons of the year, when community members perceived an increase in Illness within their communities.

I have observed that when the year starts in January, February, March, April, and May when the rain stops and the sun shines. That’s when all men and women in the community get malaria and also after a long sunny day when the rain starts to fall the people again get malaria. I have observed this since last year and last months the community was ill and even all our neighbors including father, mother, and children, and everyone was sick.—Community Member, Male

Traditional interventions for vector control and case management practices were commonly described within the communities to prevent and treat malaria. Local herbs and steam were described by many community members as the first point of ‘treatment’ prior to seeking medication prescribed at the local health facilities.

I heard [about] these from people so when my children are sick, I quickly get soursop leaves and young breadfruit leaves, boil them and give it to the children. I also tell them to get other leaves to boil and drink and whether it will help them or not. So, I think boiling and drinking herbs are good for malaria. If steam and herbs do not work after some days, I look for medicines.—Community Member, Female

Many community members reported lighting fires daily to create smoke as a way to control mosquitoes. At certain times of the year, when mosquito numbers were especially high, they added specific plant leaves—such as shade tree leaves—to the fire, believe the smoke helped repel mosquitoes. In addition to that, community members reported maintaining clean surroundings around their houses in the community to prevent mosquito breeding. As one male community member explained, *“We do clean-up of vegetation in the community, around our homes, and fill up tire tracks and holes with gravel to avoid mosquitoes from breeding.” Community Member, Male.*

#### Perceived mosquito risk and vector control tools

Community members were aware of the risk mosquitoes pose to their health. For example, the most common mosquito biting times described by the community members were early evening through till midnight, and early morning from 3am onwards. Community members also described common larval habitats for mosquitoes within their villages, including coconut shells, drains, pig wallows, stagnant water, small creeks, swamps, and tins. One male community member explained that “*Our village is in the lowlands, it’s filled with water”,* referring to rivers and creeks. He said*, "There is a big drain with water in it, and that is a home for mosquitoes”* and therefore *“It’s difficult for mosquitoes to leave us”.*

Currently, the most widely distributed vector control tool in PNG is ITNs distributed by Rotarians Against Malaria. Many participants highlighted the use and importance of ITNs for protection at night. A male community member said, *“The only way is sleeping under the bed net. I think everyone in the community sleeps under the net to prevent them from mosquito bites.”* Additionally, fogging and larvicides are deployed by private sector entities such as mining companies, in communities situated near the mines. Other vector control tools, including household aerosol insecticide sprays, such as commonly available commercial products (e.g., Mortein, manufactured by Reckitt Benckiser, UK). These products typically contain pyrethroid-based insecticides, including compounds such as allethrin and resmethrin, which are effective against mosquitoes, cockroaches, ants, and other insects. Other options such as mosquito coil repellents are also available on the market for purchase in local shops. Some participants indicated that they would procure these if funding permitted. As a male community member said, *“Coil and mortein [aerosol] if only I have money to buy, I can buy and use them.”*

Community members expressed a desire for additional support for new mosquito control tools in their communities, noting the limitations of current mosquito control measures in place and need to reduce mosquitoes present in their environments.

I think, we have tried all the home traditional vector control methods and they are not effective so I want the spray team to spray my house, and I will see if mosquitoes will continue to come or not. Community Member, Female.

#### Motivation for involvement in vector control activities

Many participants across the four communities described strong leadership, personal values, rewards and comprehensive knowledge of malaria control motivated their involvement in community-led vector control activities. As illustrated in the following quote, some community leaders and members indicated that strong leadership within a community encourages community support and motivates people to participate actively in vector control activities like environmental management.

*“The people have dug drains in the community to control the rainwater. Because when stagnant water forms in the community, it produces mosquitoes. Therefore, the community leaders talked among themselves with our brother-in-law who helped us to dig the drain.” Community Member, Female.*

Other community members described how the impact of malaria on people they care for motivated them to safeguard their families against malaria. As a male Ward Counsellor explained, *“What motivates me to support this [vector control activities] is that I have seen malaria affecting people.”*

Rewards provided to community members were a key motivator for many people to actively engage in vector control activities. These rewards could be in the form of payment and food. A male Church Leader said, *“These days I see that money or gift motivates people in the community to actively participate and take part.”* Additionally, community awareness on malaria prevention and the use of vector control tools was deemed important to empower local ownership and deeper engagement in vector control activities. The male Church leader said, *“Awareness or someone has to tell me to do it in terms of mosquito prevention methods.”*

However, despite these factors, community members described situations that discourage individuals from participating in vector control activities. This included laborious tasks such as mowing and cutting grass, digging drains and disposal pits. It was often felt that these activities do not immediately reduce mosquito density within a community and therefore do not motivate people to consistently partake in local vector control activities. As one male community member explained, *“Those who do not understand impacts and benefits [of vector control] may not actively participate.”*

Participants noted that interpersonal dynamics between community members can discourage participation in community activities. In some communities, longstanding conflicts and mistrust between individuals or families create tension and division, which may lead to reluctance to participate in community group activities. However, despite these challenges, community members generally support one another during laborious activities and put aside personal differences for the benefit of health and wellbeing.

Like now if I have grudges with someone, we have difference, you will sit with him, but I will find my own way to come see you for myself as I wanted it too…There are also others that have friendships and differences …We will say, “Forget it, the difference is between us but if it regards health, you must also come in.” So, we have such understanding there. So, the difference doesn’t affect our work. Community Member, Male

#### Affordability of vector control tools

Although community members expressed a desire for integration and implementation of additional vector control tools significant barriers may hinder progress. Participants identified key challenges including the affordability and access to new tools. Some participants reported that unless new tools were implemented as a public good through the National Department of Health, the cost of additional tools would dissuade their use. As one community member noted, existing local vector control practices—such as burning plant leaves to produce smoke to repel mosquitoes—do not cost money and are therefore preferred. When community members have to pay for additional tools, this becomes a barrier.

We understand that mosquito coil and mortein sprays are good but for most of us, where will we get the money to buy. The people who have money buy and use them, and when they do not have money, they do not use them. The best way is to use our own local repellents because we get them for free. There are many leaves that we can burn in the evening to chase mosquitoes. It does not cost money; we get them for free and burn them. When we go to bed, we sleep under the mosquito net, and we are safe. Community Member, Woman

### Factors influencing acceptability of residual spraying for community members

#### Community support for residual spraying

Many participants within the community described a desire to have residual spraying as an additional vector control tool to reduce mosquitoes and prevent sickness within the community. One participant highlighted the broader perceived benefits of residual spraying, noting the presence of other insects in their homes.

I think it is good for them to come and spray the houses because I think it will be good to kill insects like bed bugs. Maybe, the spray will also kill other insects like ants and white ants therefore, I say it’s good for the houses to be sprayed, it’s much better. Community Member, Female

This perspective illustrates how some community members viewed residual spraying as a way to address a wide range of household pests beyond mosquitoes. However, these views also reflect the potential challenge for implementation; expectations that residual spraying will kill all insects such as bedbugs and ants. Managing these expectations will be critical to the outreach and communication of any residual spraying program.

#### Enhancing protectiveness and filling a current vector control gap

Community members indicated that residual spraying would be beneficial as an additional mosquito control tool, especially when they are not under an ITN. Participants mentioned they are safe sleeping under ITNs at night, but when spending time outdoors before going under the ITNs to sleep, there was a gap in protection. It was noted that residual spraying provides additional protection against mosquitoes during those times of the evening and provides more coverage than temporary control measures such as fires.

I want to say that, when we sit around like this and discuss and tell stories, we make a fire to control mosquitoes, but we will not kill them, so when we leave the fireplace, smoke, and go out, we are not safe. Community Member, Male

#### Addressing safety concerns through community engagement

Participants across all four communities indicated their concerns about insecticide side effects. These included side effects on domestic animals such as chickens, dogs, cats and pigs. Participants feared that animals might eat the insects ‘killed’ by the spray—like cockroaches, geckos, and other insects—and become sick. For community members with health challenges such as asthma, there was particular concern about the insecticide odour on breathability. While other participants expressed concern for babies and small children who crawl on the floor and may touch the sprayed surface with the insecticide.

The children tend to eat without washing their hands, and this increases the potential risks. They will eat without washing their hands. I personally have these thoughts. They will spray the house and children might touch it... Community Member, Female

To introduce residual spraying, comprehensive awareness would be needed to describe the benefit and alleviate any community concerns on safety of the new tool.

In my opinion, this is very useful, and the condition is, there must be another awareness and get the opinions of especially the prominent people and youths and when they all agree, this (residual spraying) can come in because it is very useful. It is long-term and not for short-term to control mosquitoes. Community Member, Female

#### Past DDT spraying shapes community views on residual spraying

Some participants in the community shared their experience of historical DDT spraying campaigns used by the PNG National Malaria Control Program. Participants noted that there was a perceived increase in bedbugs within their homes following DDT spraying. With this experience came additional concern for the introduction and acceptance of new residual spraying in the PNG context. It was suggested that additional awareness must be conducted to differentiate the new residual spraying formulation from the DDT spraying previously conducted and to highlight the benefits and risks that insecticides may have on their lives.

Regarding this past experience and understanding of malaria control, there must be a good awareness about the unsafe side, or I will say everything has good and bad sides of it. So, everything needs good awareness, and people must understand and if they like they will accept and if not then they will refuse. Community Member, Male

#### Timing of communication and awareness activities

Timing and involvement of community members in communication and awareness sessions was challenging, largely due to competing priorities and the need for repeated engagement to support understanding. Deploying multiple communication and awareness strategies at different times of the day and night in the control villages was a useful approach to maximize participation. Community members also stressed that people learn and absorb information in different ways. Diversifying the resources for community awareness and ensuring culturally tailored messaging is important. A full suite of communication tools including oral storytelling sessions, locally known as ‘Tok Saves’, brochures, posters, presentations and slides are needed to ensure the full story is understood by the community.

I suggest if possible one day we can do awareness during the day and at night, we can have a slide show in the community. In our case, we can organize the community for that. I am just proposing that you can sit down with your awareness team and discuss that. Some people in terms of communication cannot understand but when they see pictures they will understand, or they will read and understand because not everyone has the same level of understanding. That’s all about my question. Community Guide, Male

For future programmatic residual spraying, communication and awareness need to be systematically designed with embedded social behaviour change practices and dedicated resources.

Yeah, but you know the information needs to reach the people clearly for them to accept the spray so they must come back to address these points, and the people must be ready to accept the spray. Our ward councillor said yes but it is good to get other people’s opinions. Community Member, Male

### Community readiness and programmatic considerations—integrating residual spraying alongside other malaria control tools

Community participants proposed a range of ideas for integrating residual spraying alongside other programmatic vector control activities and health promotion programs in PNG. Building on these perspectives, sub-national interviewees (NGO, Research, Government, implementing partners) emphasised how such tools could be operationalised within existing programs supported by the National Malaria and Vector Borne Disease Program.

#### Integrating vector control tools alongside existing health programs

Participants commonly spoke about two programs they associated with vector control in PNG. The first was the Healthy Islands Concept, a participatory community engagement, planning and monitoring approach used in Pacific Islands which was praised for its community impact, keeping communities clean and healthy through activities such as environmental management [42]. Across the four villages, many participants highlighted that environmental management was done well in their communities and encouraged the adoption by the other communities as well. One male community member said, “*Environmental management is also a good practice to prevent mosquitoes from breeding that other communities could adopt.”* Another male participant indicated that some families in the community have measures in place for environmental management, specifically disposal pits, pit toilets and disposal methods for empty tins and coconut shells.

Sub-national participants described the communal impact this program had on health, community mobilisation and social and personal responsibility. The Healthy Islands concept was described as an approach that should be adopted by the younger generations and passed on to maintain a healthy living lifestyle. However, a key acknowledgement by one participant was the mindset shift that is needed by communities, from personal health to the benefits of community health.

Changing mindsets to make sure that you have, you are using the current things for your own health. Because, if we think about personal health and then community health, and then, district, that helps us to make decisions that are correct for ourselves, for our family and then for our communities. So that's why, people use something that will have a community-wide implication as opposed to just ah you know, if I don't use it in doesn't really matter. PNG Research Partner

The second program was the ITN Distribution Program. The ITN distribution program was highlighted as a key success to the current vector control approach for PNG, particularly in reaching remote and often inaccessible communities and populations across the country. Generating the evidence needed to support the integration of new vector control tools into the ITN distribution program was encouraged by government stakeholders.

ITNs distribution, has been very successful in reaching out to the vast majority of the population, whether remote or... whatever. But I think we've succeeded through, our partners around and then to, organize this Logistics, in such a way that I've been able to reach out, to the most remote places, almost 98% level.—National Department of Health Partner

Use a different strategy or different strategies in combination. It might make a difference, but we don't have that information to be able to say that we can utilize mosquito nets together with IRS, and then things will change that. We don't know, and that is something that we, at some point, we need to get our research partners to be able to answer that question for us.—Government Partner, PNG

However, despite its success several challenges persist including the reduced bio-efficacy of mosquito nets distributed, the distribution frequency and net allocation and misuse of bed nets for alternative purposes [43]. A key informant from a national-level private sector partner organisation said, *“I’ve seen a lot of mosquito nets, say up in the highlands are being used for uh… plants, covering plants, other things not related to protection from mosquito bites here”.*

Pathways for the integration of other vector control tools into routine programs is needed. Finding effective models of implementation that leverage the existing resources, allocate resources to areas of greatest impact and reduce the barriers for uptake is important.

Slowly we are moving towards the idea of using several different vector control strategies. One of which is, of course, you know, we're trying out IRS. Hopefully, what we can do is try to map out the country and find in some places nets are more appropriate, other places, maybe IRS are not appropriate. So we just need to make decisions as to what kind of strategies we want to employ in different places and different settings.—Government Partner, PNG

## Discussion

This study provides valuable insights into community experiences of malaria and vector control tools, offering evidence to inform the prospective introduction and scale up of complementary tools such as residual spraying. The main three subthemes in the study were (i) the community experiences and drivers of vector control, (ii) factors influencing the uptake of complementary vector control tools such as residual spraying and (iii) perspectives on the integration of vector control tools within existing program efforts. This study highlights the importance of acknowledging community perspectives in shaping the success of vector control programs, and for developing locally tailored community engagement approaches in advancing malaria control both in PNG and globally.

### Community perspectives and drivers of vector control

The findings highlight that there are nuanced dynamics surrounding malaria knowledge, attitudes, and practices within communities. Community members demonstrated a solid understanding of the impact of malaria on health, productivity and seasonality alongside their treatment-seeking behaviours. However, financial barriers, unpredictable weather patterns and the laborious nature of vector control activities emerged as significant challenges, hindering effective prevention efforts.

Community members exhibited comprehensive knowledge of various vector control strategies, including ITNs, fogging, larval source management, and traditional methods such as fire. While healthcare interventions are commonly used, linking strategies like residual spraying with broader health interventions such as ongoing environmental management could drive community ownership and participation in implementation [44]. As outlined in other studies in PNG, people often pursue a combination of both biomedical and traditional solutions to health problems like malaria, thus developing strategies that consider the multi-belief system that exists around health is essential when introducing new vector control tools [45].

Community involvement in vector control activities was driven by factors such as strong leadership at both the village and household level which played a key role in motivating and organising.

community members to take part in vector control initiatives. Personal values, and the expectation of rewards, further contributed, highlighting the multifaceted nature of their motivation. These community experiences emphasised the interpersonal dynamics, such as relationships within the community which can impact an individual's behaviour in vector control. Factors like leadership and interpersonal differences were shown to either facilitate or hinder community participation in vector control efforts. For instance, strong community leadership can motivate individuals to engage in preventive actions, while interpersonal conflicts may deter participation. This has also been demonstrated in case studies around the healthy islands concept in PNG, where community ownership and participation in decision-making allowed effective accountability over the success of the concept, giving communities the incentive to mobilise and carry out implementation of activities; building pit latrines, beautifying grounds [46].

### The barriers and enablers to the introduction of residual spraying

Enablers identified in the study included a strong community desire for residual spraying, driven by the perceived benefits of controlling mosquitoes and other insects within households. This has also been experienced in Mexico where residual spraying was perceived to reduce cockroaches within households, leading to increased acceptability [47]. Community members expressed a clear preference for the continuation of residual spraying, indicating its importance in preventing sickness within the community and enhancing protectiveness when not under a mosquito net. Observed shifts in mosquito biting times in PNG alongside human sleeping behaviour reinforces the need for enhanced protection from alternative vector control tools [48]. In-depth awareness and community engagement were highlighted as crucial enablers for the successful implementation of residual spraying. Participants emphasized the need for comprehensive awareness campaigns involving prominent individuals and youth in decision-making processes could enhance community acceptance of residual spraying.

Several barriers to the uptake of residual spraying were also identified. Foremost among these was the fear of side effects, especially regarding the impact of the insecticide on domestic animals and vulnerable groups such as babies and small children. Awareness campaigns should address concerns about the safety of the insecticide used in residual spraying. Past negative experiences with DDT spraying, where some households reported increased bed bug infestations post-spraying, also contributed to community apprehension towards residual spraying. This highlights the importance of distinguishing past interventions and current strategies, as well as the need for tailored communication to address specific community concerns. Timing of communication and awareness was identified as a barrier, with some community members feeling excluded from the initial awareness sessions. It is imperative to design campaigns that are tailored to the community's needs, are conducted at different times of the day/ night and utilise preferred communication methods from that particular community. As highlighted in the results and literature, residual spraying is notoriously labour intensive, and therefore community participation is not just a matter of obtaining consent, rather integrating a diverse range of knowledge, experiences and interests into the communication strategy and intervention itself [49].

### Community readiness and programmatic considerations for the integration of vector control tools

This study provides a comprehensive overview of community perspectives on the integration of vector control tools, primarily residual spraying, with other programmatic vector control considerations and health promotion programs in PNG. Participants highlighted the importance of integrating residual spraying with existing programs, such as the ITN distribution program and the Healthy Islands Program, to enhance malaria control efforts. Factors, such as policies and resource allocation, can influence the overall effectiveness of malaria control efforts. For instance, societal attitudes towards malaria and vector control can impact community perceptions and behaviours. Leveraging existing programs and resource sharing could provide a feasible pathway towards sustainable adoption and scale up of new vector control tools [49].

Careful planning and resource allocation needs to be reviewed to ensure, the implementation of residual spraying is tailored to different contexts, scalable where needed and does not take away funding allocated to existing vector control strategies. Residual spraying requires, deep community commitment, adequate funding, trained personnel for insecticide application, sufficient supply of insecticides and logistical coordination [50], thus not all settings may be fit-for-purpose for this vector control approach. As identified by Pulford et al., the implementation of vector control in PNG can be particularly difficult in remote settings [9]. Identifying appropriate contexts with geographical accessibility is critical to the successful deployment of residual spraying in PNG.

Communities facing financial barriers may struggle to mobilize resources for effective control measures. As technology emerges for the delivery and design of new vector control tools, for example longer-lasting chemicals to reduce the spray requirements, improved aerial and hand application systems for larval source management or improved spatial emanator devices that provide protection for months, exploring different combinations of these vector control tools that account for different geographies, vector biology, malaria epidemiology and operational capacity is crucial [51].

### Study limitations

The purposive sample underpinning this study was large enough to document a range of lived experiences related to the use of vector control tools and perceptions on residual spraying in four diverse community settings. However, care must be taken not to assume that this represents all community members’ experiences of residual spraying and vector control approaches and that these findings should therefore be interpreted as contextually situated rather than broadly generalisable. Despite these limitations, internal reliability, always a concern in rigorous qualitative research, was enhanced in several ways: the team of Papua New Guinean social science researchers were well trained with wide-ranging experiences of using qualitative research methods; data was collected in local languages and during sustained periods of immersive fieldwork in each setting, which enhanced local understanding of issues and built trusted relationships with community members; interviewers worked together with study investigators throughout the study to ensure rigour and consistency in data collection and interpretation. Nevertheless, research presence and positionality, may still have influenced participants’ responses. Being reflexive about the need to dismantle power imbalances between researchers (i.e. often holding higher educational degrees, and possessing more factual knowledge of the tools under assessment) and participants (who were not always comfortable sharing their perceptions and experiences of vector control tools) helped create space for more open dialogue and supported community-led thinking about the future use of these tools in their own settings. Finally, as is always the case with qualitative research, the study captures community perceptions at a specific point in time and that the views on residual spraying and vector control tools may change in the future.

### Implications for policy and practice

The study demonstrates the prospective acceptability of residual spraying in Papua New Guinea as a complementary vector control tool, and our analyses point to several issues which could enhance the introduction of residual spraying into national policy in PNG. First, policy makers could prioritise community experiences in the design and integration of vector control interventions to ensure appropriate fit with community contexts. Acknowledging that residual spraying is more intrusive than ITN distributions, it is imperative that the design and implementation of residual spraying activities have strong community ownership and acceptability. This integration should be carefully planned, considering funding, acceptability and aligned with community practices and activities. Second, comprehensive awareness campaigns that use a multi-channel approach considering the diverse geography and communication limitations, to utilize a mix of traditional (including community gatherings and social networks) and modern (including local radio stations, TV, social media) strategies to maximise reach of messages to all population groups. Engaging community leaders, elders, and chiefs is central to enhance trust and amplify messages. Finally, a community centred approach to designing malaria control policies enables a focus on different levels of interaction; individual, interpersonal, community and societal to achieve improved implementation and adoption of new Vector control tools. Policies should address community norms and practices, societal attitudes, and resource allocation to ensure effective implementation of vector control strategies in PNG.

## Conclusion

Complementary vector control tools such as residual spraying have the potential to play an important role in malaria control efforts in PNG, to address the persisting high malaria burden. This study emphasizes the importance of understanding public attitudes, perceptions, societal norms and acceptability as new or revised interventions are introduced. Encouragingly, this study demonstrated high community acceptability to new vector control tools, particularly residual spraying, largely driven by its perceived benefits in reducing mosquito exposure and providing complementary protection alongside ITNs. However, concerns regarding insecticide safety, impacts on domestic animals, and the sustainability of implementation underscore the need for careful planning. For residual spraying and similar vector control tools to be successfully integrated into national malaria control strategies, policy development should adopt a community-centred approach that aligns with local practices and supported by adequate health system capacity.

## Supplementary Information

Supplementary Material 1.

## Data Availability

The research data are confidential and are not publicly available. Excerpts of selected transcripts have been made available within the paper. Access to the de-identified minimal dataset can be provided on reasonable request. Please contact Burnet Institute’s Research Governance Office on [research.governance@burnet.edu.au] (mailto: research.governance@burnet.edu.au).

## Abbreviations

ITNs: Insecticide Treated Nets
VCTs: Vector Control Tools
DDT: Dichlorodiphenyltrichloroethane
SEM: Socio-ecological model
RAM: Rotary Against Malaria
PNG: Papua New Guinea
PNGIMR: Papua New Guinea Institute of Medical Research
TV: Television
RS: Residual Spraying
NATNAT: Newly Adapted Tools and Network Against Mosquito-Borne Disease Transmission

## References

[1] World Malaria Report 2025: addressing the threat of antimalarial drug resistance. World Health Organization; 2025.

[2] Farquhar R, Dori A, MacCana S, Tefuarani N, Lavu E, Barry A, et al. STRIVE PNG: using a partnership-based approach in implementation research to strengthen surveillance and health systems in Papua New Guinea. Health Res Policy Syst. 2022 Dec 1;20(1). 10.1186/s12961-022-00840-3 PMC8976436 35366903

[3] Vinit R, Timinao L, Bubun N, Katusele M, Robinson LJ, Kaman P, et al. Decreased bioefficacy of long-lasting insecticidal nets and the resurgence of malaria in Papua New Guinea. Nat Commun. 2020;11(1). 10.1038/s41467-020-17456-2 PMC7371689 32686679

[4] Hetzel MW, Morris H, Tarongka N, Barnadas C, Pulford J, Makita L, et al. Prevalence of malaria across Papua New Guinea after initial roll-out of insecticide-treated mosquito nets. Tropical Med Int Health. 2015;20(12):1745–55. 10.1111/tmi.12616 26427024

[5] Hetzel MW, Gideon G, Lote N, Makita L, Siba PM, Mueller I. Ownership and usage of mosquito nets after four years of large-scale free distribution in Papua New Guinea. Malar J. 2012;11. 10.1186/1475-2875-11-192 PMC3422192 22682111

[6] Pulford J, Oakiva T, Angwin A, Bryant M, Mueller I, Hetzel MW. Indifferent to disease: A qualitative investigation of the reasons why some Papua New Guineans who own mosquito nets choose not to use them. Soc Sci Med [Internet]. 2012 Dec 1 [cited 2025 Dec 15];75(12):2283–90. Available from: https://www.sciencedirect.com/science/article/abs/pii/S0277953612006375 10.1016/j.socscimed.2012.08.030 22995668

[7] Rodríguez-Rodríguez D, Katusele M, Auwun A, Marem M, Robinson LJ, Laman M, et al. Human behavior, livelihood, and malaria transmission in two sites of Papua New Guinea. J Infect Dis. 2021;223:S171–86. 33906224 10.1093/infdis/jiaa402 PMC8079136

[8] Keven JB, Katusele M, Vinit R, Rodríguez-Rodríguez D, Hetzel MW, Robinson LJ, et al. Vector composition, abundance, biting patterns and malaria transmission intensity in Madang, Papua New Guinea: assessment after 7 years of an LLIN-based malaria control programme. Malar J. 2022 Dec 1;21(1). 10.1186/s12936-021-04030-4 PMC8729043 34983530

[9] Pulford J, Tandrapah A, Atkinson JA, Kaupa B, Russell T, Hetzel MW. Feasibility and acceptability of insecticide-treated plastic sheeting (ITPS) for vector control in Papua New Guinea [Internet]. 2012. Available from: http://www.malariajournal.com/content/11/1/342 10.1186/1475-2875-11-342 PMC3507715 23046535

[10] Kuadima JJ, Timinao L, Naidi L, Tandrapah A, Hetzel MW, Czeher C, et al. Long-term acceptability, durability and bio-efficacy of ZeroVector® durable lining for vector control in Papua New Guinea. Malar J. 2017;16(1):93. 10.1186/s12936-017-1742-y PMC5329951 28241875

[11] Martínez PM, Gabong R, Balanza N, Luana S, Sanz S, Raulo S, et al. Coverage, Determinants of Use and Repurposing of Long-Lasting Insecticidal Nets Two Years After a Mass Distribution in Lihir Islands, Papua New Guinea: A Cross-Sectional Study. 2021 May 21 [cited 2025 Dec 15]; Available from: https://www.researchsquare.com 10.1186/s12936-021-03867-z PMC8336363 34348727

[12] Health Organization W. An operational manual for indoor residual spraying (IRS) for malaria transmission control and elimination second edition indoor residual spraying.

[13] Munn Z, Stone JC, Barker TH, Price C, Pollock D, Kabaghe AN, et al. Residual insecticide surface treatment for preventing malaria: a systematic review protocol. Vol. 12, Systematic Reviews. BioMed Central Ltd; 2023. 10.1186/s13643-023-02259-5 PMC10233908 37264462

[14] Gobena T, Berhane Y, Worku A. Women’s knowledge and perceptions of malaria and use of malaria vector control interventions in Kersa, Eastern Ethiopia. Glob Health Action. 2013;6(1). 10.3402/gha.v6i0.20461 PMC3664061 23706162

[15] Ediau M, Babirye JN, Tumwesigye NM, Matovu JK, MacHingaidze S, Okui O, et al. Community knowledge and perceptions about indoor residual spraying for malaria prevention in Soroti district, Uganda: A cross-sectional study. Malar J. 2013;12(1). 10.1186/1475-2875-12-170 PMC3679793 23705591

[16] Keven JB, Henry-Halldin CN, Thomsen EK, Mueller I, Siba PM, Zimmerman PA, et al. Pyrethroid susceptibility in natural populations of the anopheles punctulatus group (Diptera: Culicidae) in Papua New Guinea. Am J Trop Med Hyg. 2010;83(6):1259–61. 21118931 10.4269/ajtmh.2010.10-0422 PMC2990041

[17] Parkinson A. Malaria in Papua New Guinea 1973. 1973 [cited 2025 Jul 4]; Available from: 10.5555/19750521516

[18] Cleary E, Hetzel MW, Clements ACA. A review of malaria epidemiology and control in Papua New Guinea 1900 to 2021: Progress made and future directions. Front Epidemiol. 1900;2:980795. 10.3389/fepid.2022.980795 PMC10910954 38455277

[19] Malaria In Papua New Guinea 1973-compressed (1).

[20] Gunither JT. Malaria Scandal. 1971;28:2.

[21] Hetzel MW, Reimer LJ, Gideon G, Koimbu G, Barnadas C, Makita L, et al. Changes in malaria burden and transmission in sentinel sites after the roll-out of long-lasting insecticidal nets in Papua New Guinea. Parasit Vectors. 2016;9(1). 10.1186/s13071-016-1635-x PMC4908799 27301964

[22] Swai JK, Moore SJ. Beyond repellents: spatial emanators for the control of malaria in Africa. Lancet. 2025;405(10473):101–3. 39709978 10.1016/S0140-6736(24)02754-5

[23] Liverani M, Charlwood JD, Lawford H, Yeung S. Field assessment of a novel spatial repellent for malaria control: A feasibility and acceptability study in Mondulkiri, Cambodia. Malar J [Internet]. 2017 Oct 13 [cited 2025 Jul 8];16(1):1–12. Available from: 10.1186/s12936-017-2059-6 10.1186/s12936-017-2059-6 PMC5640900 29029614

[24] Ogoma SB, Mmando AS, Swai JK, Horstmann S, Malone D, Killeen GF. A low technology emanator treated with the volatile pyrethroid transfluthrin confers long term protection against outdoor biting vectors of lymphatic filariasis, arboviruses and malaria. PLoS Negl Trop Dis [Internet]. 2017 Apr 7 [cited 2025 Jul 8];11(4):e0005455. Available from: 10.1371/journal.pntd.0005455 10.1371/journal.pntd.0005455 PMC5384659 28388682

[25] García GA, Fuseini G, Donfack OT, Wofford RN, Nlang JAM, Efiri PB, et al. The need for larval source management accompanying urban development projects in malaria endemic areas: a case study on Bioko Island. Malar J [Internet]. 2022 Dec 1 [cited 2025 Jul 8];21(1):1–9. Available from: 10.1186/s12936-022-04362-9 10.1186/s12936-022-04362-9 PMC9664620 36376966

[26] Okumu F, Moore SJ, Selvaraj P, Yafin AH, Juma EO, Shirima GSG, et al. Elevating larval source management as a key strategy for controlling malaria and other vector-borne diseases in Africa. Vol. 18, Parasites and Vectors . BioMed Central Ltd; 2025. 10.1186/s13071-024-06621-x PMC11803969 39915825

[27] Kamndaya M, Mfipa D, Lungu K. Household knowledge, perceptions and practices of mosquito larval source management for malaria prevention and control in Mwanza district, Malawi: a cross‐sectional study. Malar J [Internet]. 2021 Dec 1 [cited 2025 Jul 8];20(1):1–8. Available from: 10.1186/s12936-021-03683-5 10.1186/s12936-021-03683-5 PMC7967974 33731146

[28] Millat-Martínez P, Katusele M, Kasian B, Omera E, Jamea E, Lorry L, et al. Human and entomological determinants of malaria transmission in the Lihir Islands of Papua New Guinea: A cross-sectional study. PLoS Negl Trop Dis [Internet]. 2025 Jan 1 [cited 2025 Jul 8];19(1):e0012277. Available from: 10.1371/journal.pntd.0012277 10.1371/journal.pntd.0012277 PMC11734946 39752628

[29] Bangs MJ. Sustainable Investment in Extractive Industry Development: Implementation of Vector-borne Disease and Pest Control for Workforce Protection in Papua New Guinea. Society of Petroleum Engineers - SPE/APPEA Int Conference on Health, Safety and Environment in Oil and Gas Exploration and Production 2012: Protecting People and the Environment - Evolving Challenges [Internet]. 2012 Sep 11 [cited 2025 Dec 15];3:2604–14. Available from: 10.2118/157662-MS

[30] Rivera EP, Arrivillaga MR, Juárez JG, De Urioste-Stone SM, Berganza E, Pennington PM. Adoption of community-based strategies for sustainable vector control and prevention. BMC Public Health [Internet]. 2023 Dec 1 [cited 2025 Jul 8];23(1):1–15. Available from: 10.1186/s12889-023-16516-8 10.1186/s12889-023-16516-8 PMC10512496 37730592

[31] Atkinson JAM, Fitzgerald L, Toaliu H, Taleo G, Tynan A, Whittaker M, et al. Community participation for malaria elimination in Tafea Province, Vanuatu: Part I. Maintaining motivation for prevention practices in the context of disappearing disease. Malar J. 2010;9:93. 10.1186/1475-2875-9-93 PMC2873527 20380748

[32] XYZ Act 1998: Elizabeth II. Chapter 9999. Stationery Office; 1998.

[33] Atkinson JA, Vallely A, Fitzgerald L, Whittaker M, Tanner M. The architecture and effect of participation: A systematic review of community participation for communicable disease control and elimination. Implications for malaria elimination. Malaria J. 2011;10. 10.1186/1475-2875-10-225 PMC3171376 21816085

[34] Hii J, Hustedt J, Bangs MJ. Residual Malaria Transmission in Select Countries of Asia-Pacific Region: Old Wine in a New Barrel. Journal of Infectious Diseases [Internet]. 2021 May 1 [cited 2025 Jul 8];223(12 Suppl 2): S111–42. Available from: https://pubmed.ncbi.nlm.nih.gov/33906222/10.1093/infdis/jiab004 PMC8079134 33906222

[35] Munguambe K, Pool R, Montgomery C, Bavo C, Nhacolo A, Fiosse L, et al. What drives community adherence to indoor residual spraying (IRS) against malaria in Manhiça district, rural Mozambique: A qualitative study. Malar J [Internet]. 2011 Nov 23 [cited 2025 Jul 8];10(1):1–13. Available from: 10.1186/1475-2875-10-344 10.1186/1475-2875-10-344 PMC3339361 22111698

[36] Olmos-Vega FM, Stalmeijer RE, Varpio L, Kahlke R. A practical guide to reflexivity in qualitative research: AMEE Guide No. 149. Med Teach. 2023;45(3):241–51. 10.1080/0142159X.2022.2057287 35389310

[37] Bell SA. Monitoring and evaluation in health and social development. Interpretive and ethnographic perspectives [Internet]. 2016. Available from: https://www.researchgate.net/publication/299713347

[38] Farquhar RJ, Kerry Z, Mohamed Y, Morgan C, Dori A, McEwen S, et al. Baseline assessment of front-line health system capacity in vector-borne disease surveillance and response in Papua New Guinea. PLOS Global Public Health. 2025 Apr 1;5(4 APRIL). 10.1371/journal.pgph.0004108 PMC12043147 40305556

[39] Dori A, Farquhar R, Kelebi T, Waipeli E, Kerry Z, Ruybal-Pesántez S, et al. Partnership-Based Approach to Infectious Disease Research in Papua New Guinea. In: Sustainable Development Goals Series. Springer; 2024. p. 133–46.

[40] (PDF) Comparison of Convenience Sampling and Purposive Sampling [Internet]. [cited 2025 Sep 18]. Available from: https://www.academia.edu/35915814/Comparison_of_Convenience_Sampling_and_Purposive_Sampling

[41] Braun V, Clarke V. Using thematic analysis in psychology. Qual Res Psychol. 2006;3(2):77–101.

[42] Yeung S, Selep J. Healthy Islands Concept in Papua New Guinea. 2016.

[43] Hetzel MW. An integrated approach to malaria control in Papua New Guinea [Internet]. 2014. Available from: https://www.researchgate.net/publication/49652943 21125984

[44] Raghavendra K, Barik TK, Reddy BPN, Sharma P, Dash AP. Malaria vector control: From past to future. Parasitol Res [Internet]. 2011 Apr [cited 2025 Jul 8];108(4):757–79. Available from: https://pubmed.ncbi.nlm.nih.gov/21229263/10.1007/s00436-010-2232-0 21229263

[45] Andrew EVW, Pell C, Angwin A, Auwun A, Daniels J, Mueller I, et al. Knowledge, attitudes, and practices concerning malaria in pregnancy: Results from a qualitative study in Madang, Papua New Guinea. PLoS One. 2015 Apr 20;10(4). 10.1371/journal.pone.0119077 PMC4404357 25893405

[46] Community Engagement Key Function Committee Task Force C. PR I NC I PL E S OF COMMUNITY ENGAGEMENT Clinical and Translational Science Awards Consortium Community Engagement Key Function Committee Task Force on the Principles of Community Engagement.

[47] Rodríguez AD, Penilla RP, Rodríguez MH, Hemingway J, Trejo A, Hernández-Avila JE. Acceptability and perceived side effects of insecticide indoor residual spraying under different resistance management strategies. Salud Publica Mex [Internet]. 2006 [cited 2025 Jul 8];48(4):317–24. Available from: https://pubmed.ncbi.nlm.nih.gov/16913456/10.1590/s0036-36342006000400006 16913456

[48] Thomsen EK, Koimbu G, Pulford J, Jamea-Maiasa S, Ura Y, Keven JB, et al. Mosquito behavior change after distribution of bednets results in decreased protection against malaria exposure. In: Journal of Infectious Diseases. Oxford University Press; 2017. p. 790–7. 10.1093/infdis/jiw615 PMC5388271 28007921

[49] Bartumeus F, Costa GB, Eritja R, Kelly AH, Finda M, Lezaun J, et al. Sustainable innovation in vector control requires strong partnerships with communities. PLoS Negl Trop Dis [Internet]. 2019 [cited 2025 Jul 8];13(4):e0007204. Available from: 10.1371/journal.pntd.0007204 10.1371/journal.pntd.0007204 PMC6483154 31022178

[50] Obeagu EI, Obeagu GU. Emerging public health strategies in malaria control: innovations and implications. Ann Med Surgery. 2024;86(11):6576–84. 10.1097/MS9.0000000000002578 PMC11543165 39525724

[51] Killeen GF, Tatarsky A, Diabate A, Chaccour CJ, Marshall JM, Okumu FO, et al. Developing an expanded vector control toolbox for malaria elimination. BMJ Glob Health [Internet]. 2017 [cited 2025 Jul 8];2(2). Available from: https://pubmed.ncbi.nlm.nih.gov/28589022/10.1136/bmjgh-2016-000211 PMC5444090 28589022

